# Population attributable fraction: comparison of two mathematical procedures to estimate the annual attributable number of deaths

**DOI:** 10.1186/1742-5573-7-8

**Published:** 2010-08-31

**Authors:** Bernard CK Choi

**Affiliations:** 1 Centre for Chronic Disease Prevention and Control, Public Health Agency of Canada (PHAC), Government of Canada, Ottawa, Ontario, Canada; 2 Department of Epidemiology and Community Medicine, University of Ottawa, Ottawa, Ontario, Canada; 3 Dalla Lana School of Public Health, University of Toronto, Toronto, Ontario, Canada; 4 Shantou University Medical College, Shantou, China

## Abstract

**Objective:**

The purpose of this paper was to compare two mathematical procedures to estimate the annual attributable number of deaths (the Allison et al procedure and the Mokdad et al procedure), and derive a new procedure that combines the best aspects of both procedures. The new procedure calculates attributable number of deaths along a continuum (i.e. for each unit of exposure), and allows for one or more neutral (neither exposed nor nonexposed) exposure categories.

**Methods:**

Mathematical derivations and real datasets were used to demonstrate the theoretical relationship and practical differences between the two procedures. Results of the comparison were used to develop a new procedure that combines the best features of both.

**Findings:**

The Allison procedure is complex because it directly estimates the number of attributable deaths. This necessitates calculation of probabilities of death. The Mokdad procedure is simpler because it estimates the number of attributable deaths indirectly through population attributable fractions. The probabilities of death cancel out in the numerator and denominator of the fractions. However, the Mokdad procedure is not applicable when a neutral exposure category exists.

**Conclusion:**

By combining the innovation of the Allison procedure (allowing for a neutral category) and the simplicity of the Mokdad procedure (using population attributable fractions), this paper proposes a new procedure to calculate attributable numbers of death.

## Background

There are two mathematical procedures to estimate the number of deaths attributable to a risk factor such as obesity, smoking or alcohol consumption. Number of attributable deaths is the number of deaths in a population that could be avoided if the effects of the risk factor were eliminated from the population. The two procedures are Allison et al [1] and Mokdad et al [2]. Both procedures are under the assumptions of no confounding and no effect modification. Both can be applied to risk factors with polytomous exposure categories.

The Allison procedure [1], originally developed for obesity attributable deaths, is rather complex. It involves 12 steps, uses hazard ratios, and requires calculating hazard rates by using a mathematical process to solve for an unknown quantity. The Mokdad procedure [2] on the other hand, is simpler. It involves only 6 steps, uses relative risks, and does not require solving for any unknown quantity.

In general, a common belief is that the more complex the procedure, the more accurate the results. Allison et al further stipulated that their procedure accounts for "complications", because it can estimate attributable deaths for body mass index (BMI) along a continuum (ie, for each unit of BMI), and can adjust for time using hazard ratio (HR) that the relative risks (RR) cannot achieve [1]. As a result, Mokdad et al used the Mokdad procedure to estimate the attributable numbers for tobacco and alcohol, and then they reverted to the more complex Allison procedure to estimate the attributable number for obesity.

A detailed read of Allison et al's paper revealed that two steps in the Allison procedure are not well-documented. First, while the equation for the overall number of deaths attributable to obesity and overweight (ω) is given in their paper, the equation to calculate the number of deaths attributable to each individual BMI category is missing. It is therefore unclear how data in their table three can lead to results in their table four. Second, it is said in their paper that the hazard (λ) can be obtained by numerically solving a complex equation for λ. Τhe actual method, however, is not given. For the less sophisticated users, the Allison procedure is not user-friendly.

There are several questions arising from looking at these two procedures: How are the Allison and Mokdad procedures mathematically and practically different? What are the best aspects of each procedure? Can the underlying equations be combined or modified to take advantage of the best aspects of both?

This paper compares the Allison and Mokdad procedures for the estimation of annual attributable number of deaths, both mathematically and using real data. The paper also "recovers" the missing Allison equation to calculate the individual number of deaths attributable to each BMI category, develops a similar and simpler equation using the logic of the Mokdad procedure, compares estimated number of attributable deaths under the HR and RR models, and looks at several options for numerically solving the equation for λ. This paper also proposes a modified Mokdad procedure that can achieve the same results as the Allison procedure.

## Methods

Mathematical derivations from first principles from population attributable fraction (PAF), defined as the proportion of deaths in a population that can be attributed to the causal effects of a risk factor or set of factors, were used to demonstrate the relationship and differences of the Allison and Mokdad procedures. The missing mathematical equation to estimate the number of deaths attributable to each exposure category was derived for the Allison procedure. A similar equation was created for the Mokdad procedure. The two procedures were then "taken apart" and the logics behind the two procedures were examined and compared. Based on this, a new procedure (modified Mokdad) was developed combining the innovation of the Allison procedure and the logic of the Mokdad procedure. Finally, estimation methods under the hazard ratio and relative risk models were compared. Some options for solving for λ were described. Real datasets provided by Allison et al were used to illustrate the practical differences of the two procedures (Allison and the new procedure provided in this paper).

## Results

Table 1 is a conversion table of the notations used in Allison et al, Mokdad et al, and this paper. The 12 steps in the Allison Procedure and the 6 steps in the Mokdad procedure to calculate the attributable number of deaths are summarized in Additional file 1, Appendix S1, using their original notations.

**Table 1:** Conversion table of notations.

Variable	***Allison et al ***[1]	***Mokdad et al ***[2]	*This Paper*
No. of deaths attributable	ω	ω	ω
Total no. of deaths in population	M	M	M
Total no. in population	N		N
Fraction of population nonexposed	P(R)	P_0_	f_0_, 1- Σf_i,_ 1- Σf_i _- f_q_
Fraction of population exposed	P(O)	Σ P_i_	f, Σf_i_
Fraction of population exposed to an exposure category (i)		P_i_	f_i_
Fraction of population exposed to a neutral category (e.g., underweight)	P(Q)		f_q_
Hazard ratio (HR) for an exposure category	h		h
Hazard ratio (HR) for a neutral category (e.g., underweight)	q		q
Probability of death in a year in population	P(D), M/N		p, P(D)
Hazard of death in the nonexposed	λ		λ
Conditional probability of death in a year in nonexposed	P(D|R), 1 - e^-λ^		p_0_, P(D|E_0_)
Conditional probability of death in a year in various exposure categories	P(D|O), 1 - e^-hλ^		R_i _p_0_
Conditional probability of death in a year in a neutral category (e.g., underweight)	P(D|Q), 1 - e^-qλ^		p_q_
Relative risk	RR, P(D|O)/P(D|R)	RR_i_	R_i_
Population attributable fraction		PAF	PAF

### 1. Mathematical proof that the Allison procedure and Mokdad procedure differ in a neutral exposure category (Q)

Based on Levin [3], and using notations in Table 2,

**Table 2:** Distributions according to death and an exposure variable: Dichotomous exposure

	Nonexposed E_0_	Exposed E	Total
Death D	(1 - f) p_0_	f R p_0_	p
No death $\bar{D}$	(1 - f) (1 - p_0_)	f (1 - R p_0_)	1 - p
Total	1 - f	f	1

Let

p = probability of death in population

f = fraction of population exposed

p_0 _= probability of death in the nonexposed

R = relative risk

It follows that for a dichotomous exposure variable, based on the second row of the table,

$$\text{p}=\left(\text{1}–\text{f}\right){\text{ p}}_{0}+{\text{f R p}}_{0}$$

$$\text{PAF}=\frac{\text{p}-{\text{p}}_{0}}{\text{p}}$$

where PAF is population attributable fraction; p is the probability of death in the population, or P(D); p_0 _is the probability of death among the nonexposed, or P(D|E_0_). Then using equation T1 (Table 2),

$$\begin{array}{l} PAF=\frac{p-p_{0}}{p}=\frac{(1-f)\;p_{0}+f\;R\;p_{0}-p_{0}}{(1-f)\;p_{0}+f\;Rp_{0}}\\ =\frac{p_{0}-fp_{0}+fR\;p_{0}-p_{0}}{p_{0}-fp_{0}+fR\;p_{0}}=\frac{f\;R-f}{1+f\;R-f}\\ \text{PAF}=\frac{\text{f}\;(\text{R}\;\text{-}\;1)}{1+\text{f}\;(\text{R}\;\text{-}\;1)} \end{array}$$

Equation 2 is another frequently quoted form of PAF [3].

Extending the same methodology above for the case of a dichotomous exposure variable to the case of a polytomous exposure variable, and using equation T2 (Table 3),

**Table 3:** Distributions according to death and an exposure variable: Polytomous exposure

	Nonexposed E_0_	Exposed Categories E_1_...E_i_...E_k_	Total
Death D	(1 - Σf_i_) p_0_	Σ f_i _R_i _p_0_	P
No death $\bar{D}$	(1 - Σf_i_) (1-p_0_)	Σ f_i _(1- R_i _p_0_)	1-p
Total	1 - Σf	Σf_i_	1

Let

f_i _= fraction of population exposed to exposure category i

R_i _= relative risk for exposure category i compared to non exposed

It follows that for a polytomous exposure variable

$$\text{p}=(\text{1}–\Sigma {\text{f}}_{\text{i}}){\text{ p}}_{0}+\Sigma {\text{f}}_{\text{i}}{\text{R}}_{\text{i}}{\text{p}}_{0}$$

$$\text{PAF}=\frac{{\text{p-p}}_{0}}{\text{p}}=\frac{(1\text{-}\sum {\text{f}}_{\text{i}}){\text{p}}_{0}+\sum {\text{f}}_{\text{i}}{\text{ R}}_{\text{i}}{\text{ p}}_{0}{\text{-p}}_{0}}{(1\text{-}\sum {\text{f}}_{\text{i}}){\text{p}}_{0}+\sum {\text{f}}_{\text{i}}\;{\text{R}}_{\text{i}}\;{\text{p}}_{0}}$$

$$\text{PAF}=\frac{(1\text{-}\sum {\text{f}}_{\text{i}})+\sum {\text{f}}_{\text{i}}{\text{ R}}_{\text{i}}\text{-}1}{(1\text{-}\sum {\text{f}}_{\text{i}})+\sum {\text{f}}_{\text{i}}\;{\text{R}}_{\text{i}}}$$

Equation 4 is Mokdad et al [2] (see Additional file 1, Appendix S1, equation A3), as 1-∑f_i _is P_0 _(Table 1).

From equation 3,

$$\begin{array}{l} \text{PAF}=\frac{(1\text{-}\sum {\text{f}}_{\text{i}}){\text{p}}_{0}+\sum {\text{f}}_{\text{i}}\;{\text{R}}_{\text{i}}{\text{ p}}_{0}\text{-}\left[(1\text{-}\sum {\text{f}}_{\text{i}}){\text{p}}_{0}+\sum {\text{f}}_{\text{i}}\;{\text{p}}_{0}\right]}{(1\text{-}\sum {\text{f}}_{\text{i}}){\text{p}}_{0}+\sum {\text{f}}_{\text{i}}\;{\text{R}}_{\text{i}}{\text{ p}}_{0}}\\ =1-\frac{(1\text{-}\sum {\text{f}}_{\text{i}}){\text{p}}_{0}+\sum {\text{f}}_{\text{i}}\;{\text{p}}_{0}}{(1\text{-}\sum {\text{f}}_{\text{i}}){\text{p}}_{0}+\sum {\text{f}}_{\text{i}}{\text{ R}}_{\text{i}}{\text{ p}}_{0}} \end{array}$$

But because p = (1-∑f_i_)p_0 _+ ∑f_i _R_i _p_0_, see equation T2 in Table 3, therefore

$$\text{PAF}\;\text{=}\;\text{1-}\frac{1}{\text{p}}\;\left[(1\text{-}\sum {\text{f}}_{\text{i}}){\text{p}}_{0}+\sum {\text{f}}_{\text{i}}\;{\text{p}}_{0}\right]$$

Equation 5 is a modified form of Allison et al [1] (see Additional file 1, Appendix S1, equation A1), as shown below. From equations A2 (Additional file 1, Appendix S1) and 5, and given p = M/N,

$$\begin{array}{l} \omega =\text{M}\cdot \text{PAF}\\ =\text{M}\left\{1-\frac{1}{\text{p}}\left[(1-\sum {\text{f}}_{\text{i}}){\text{p}}_{0}+\sum {\text{f}}_{\text{i}}{\text{p}}_{0}\right]\right\}\\ =\text{M}-\text{N}[(1-\Sigma {\text{f}}_{\text{i}})\text{p0}+\Sigma {\text{f}}_{\text{i}}\text{p0}]\\ =\text{M}-\text{N}({\text{f}}_{0}\text{p0}+\Sigma {\text{f}}_{\text{i}}\text{p0})\\ =\text{M}-\text{N}\;[\text{P}(\text{R})\text{P}(\text{D}\;|\;\text{R})+\text{P}(\text{O})\text{P}(\text{D}\;|\;\text{R})] \end{array}$$

Comparing the Mokdad equation (6) with the Allison equation (A1), the Mokdad procedure allows only for a single nonexposed category E_0 _and a number of exposure categories E_1 _... E_i _...E_k _(see Table 3), while the Allison procedure in addition allows for a neutral (i.e., neither nonexposed nor exposed) category, in this case the category Q (underweight) (see Table 4).

**Table 4:** Consideration of a neutral exposure category

	Nonexposed E_0 _ (e.g. normal weight, R)	Exposed Categories E_1_...E_i_...E_k _ (e.g. overweight and obese, O)	Neutral Category (e.g. underweight, Q)	Total
Death D	(1 - Σf_i_-f_q_) p_0_	Σf_i _R_i _p_0_	f_q _p_q_	p
No death $\bar{D}$	(1 - Σf_i_- f_q_) (1 - p_0_)	Σf_i_(1- R_i _p_0_)	f_q_(1-p_q_)	1 - p
Total	1 - Σf_i_-f_q_	Σf_i_	f_q_	1

Let

f_0 _= fraction of population nonexposed = 1 -Σf_i _- f_q_

f_q _= fraction of population underweight

It follows that when a neutral category is involved

$$\begin{array}{l} \text{p}={\text{f}}_{0}{\text{p}}_{0}+\Sigma {\text{f}}_{\text{i}}{\text{R}}_{\text{i}}{\text{p}}_{0}+{\text{f}}_{\text{q}}{\text{p}}_{\text{q}}\\ =(\text{1}–\Sigma {\text{f}}_{\text{i}}–{\text{f}}_{\text{q}}){\text{ p}}_{0}+\Sigma {\text{f}}_{\text{i}}{\text{R}}_{\text{i}}{\text{p}}_{0}+{\text{f}}_{\text{q}}{\text{p}}_{\text{q}} \end{array}$$

The methodology of Allison et al leads to a modified Levin equation as shown below.

The original Allison equation (Additional file 1, Appendix S1, equation A1) was written for a three category exposure (R, reference group or the nonexposed E_0_; O, overweight and obese groups; and Q, the underweight group) (Table 4) and is

ω=M−N[P(R)P(D|R)+P(O)P(D|R)+P(Q)P(D|Q)]

where ω is number of deaths attributable to exposure categories E_1 _... E_i _... E_k _combined, M is total number of deaths, N is total number in population, P(R) is f_0 _= 1- Σf_i _- f_q _, P(O) is Σf_i_, P(D|R) is p_0_.

From equation A1, and using the notations of this paper (Table 4),

$$\begin{array}{l} \omega =\text{ M }–\text{ N }({\text{f}}_{0}{\text{p}}_{0}+\Sigma {\text{f}}_{\text{i}}{\text{p}}_{0}+{\text{ f}}_{\text{q}}{\text{p}}_{\text{q}})\\ \;=\text{M}-\text{N}[(1-\Sigma {\text{f}}_{\text{i}}-{\text{f}}_{\text{q}}){\text{p}}_{0}+\Sigma {\text{f}}_{\text{i}}{\text{p}}_{0}+{\text{f}}_{\text{q}}{\text{p}}_{\text{q}}]\\ =\text{M}\left\{1-\frac{1}{\text{p}}\left[(1-\sum {\text{f}}_{\text{i}}-{\text{f}}_{\text{q}}){\text{p}}_{0}+\sum {\text{f}}_{\text{i}}{\text{p}}_{0}+{\text{f}}_{\text{q}}{\text{p}}_{\text{q}}\right]\right\} \end{array}$$

given p = M/N. And since PAF= ω/M (equation A2),

$$\begin{array}{c} \text{PAF}=1-\frac{1}{\text{p}}\left[(1-\sum {\text{f}}_{\text{i}}-{\text{f}}_{\text{q}}){\text{p}}_{0}+\sum {\text{f}}_{\text{i}}{\text{p}}_{0}+{\text{f}}_{\text{q}}{\text{p}}_{\text{q}}\right]\\ =\frac{\text{p-}\left[(1\text{-}\sum {\text{f}}_{\text{i}}{\text{-f}}_{\text{q}}){\text{p}}_{0}+\sum {\text{f}}_{\text{i}}{\text{p}}_{0}+{\text{f}}_{\text{q}}{\text{p}}_{\text{q}}\right]}{\text{p}}\\ =\frac{\text{p-}(1{\text{-f}}_{\text{q}}){\text{p}}_{0}-{\text{f}}_{\text{q}}{\text{p}}_{\text{q}}}{\text{p}} \end{array}$$

Comparing equation 8 (derived from Allison et al) with Levin's original equation for PAF (equation 1) which is identical to the Mokdad procedure, the Allison procedure subtracts out a certain weighted proportion of deaths associated with the neutral category (the underweight Q) from the attributable deaths to the exposure (the overweight and obese). In other words, the Allison procedure allows for a neutral exposure category (neither nonexposed nor exposed), while the Mokdad procedure does not.

### 2. Recovery of the Allison equation for number of deaths attributable to each exposure category

To derive the missing Allison equation, from equation A1,

ω= M-N[P(R)P(D | R)+P(O)P(D | R)+P(Q)P(D | Q)]=M-N[P(R)P(D | R)+ΣP(Oi)P(D | R)+P(Q)P(D | Q)]=N[P(R)P(D | R)+P(O)P(D | O)+ P(Q)P(D | Q)]  –N[P(R)P(D | R)+P(O)P(D | R)+P(Q)P(D | Q)]=N[P(O)P(D | O)–P(O)P(D | R)]=N P(O)[P(D | O)–P(D | R)]= N Σ{P(Oi) [P(D | Oi)–P(D | R)]}

Τherefore, the missing equation is

$$\omega_{\text{i}}=\text{ N P}({\text{O}}_{\text{i}})\;[\text{P}(\text{D}\;|\;{\text{O}}_{\text{i}})-\text{P}\left(\text{D}\;|\;\text{R}\right)]$$

where ω_i _is number of deaths attributable to each exposure category i.

Equations 9 and 10 are new equations we created for the Allison procedure. It is worth noting that the underweight (Q) category disappears in equations 9 and 10, as it does not play a role in the calculations of attributable numbers to obesity (O). However, equations 9 and 10 are still difficult to use because the probabilities of death in the exposed and reference groups can only be estimated through a complex process:

$$\text{P}(\text{D}\;|\;{\text{O}}_{\text{i}})=\text{1}–{\text{e}}^{-\text{h}\lambda },\text{ P}\left(\text{D}\;|\;\text{R}\right)=\text{1}–{\text{e}}^{-\lambda }.$$

The next section shows that the Mokdad procedure can be modified to do the same calculations as the Allison procedure, but more simply.

### 3. Development of an equation for number of deaths attributable to each exposure category for the modified Mokdad procedure

The Allison procedure directly estimates the number of attributable deaths, ω. The Mokdad procedure indirectly estimates ω by first estimating PAF. Using the logic of the Mokdad procedure, we develop a new, modified Mokdad procedure to estimate ω as follows:

From equation 10, and using notations in Table 4,

$$\begin{array}{l} \omega_{\text{i}}=\text{N P}({\text{O}}_{\text{i}})\;[\text{P}(\text{D}\;|\;{\text{O}}_{\text{i}})–\text{P}\left(\text{D}\;|\;\text{R}\right)]\\ \text{=N P}({\text{O}}_{\text{i}})\;[{\text{RR}}_{\text{i}}\text{P}\left(\text{D}\;|\;\text{R}\right)–\text{P}\left(\text{D}\;|\;\text{R}\right)]\;\\ {\text{=N f}}_{\text{i}}[{\text{R}}_{\text{i}}{\text{p}}_{0}–{\text{p}}_{0}]\;={\text{ N f}}_{\text{i}}({\text{R}}_{\text{i}}–\text{1}){\text{ p}}_{0} \end{array}$$

$$\begin{array}{l} \text{M}=\text{N p}=\text{N }({\text{f}}_{0}{\text{p}}_{0}+\Sigma {\text{f}}_{\text{i}}{\text{R}}_{\text{i}}{\text{p}}_{0}+{\text{f}}_{\text{q}}{\text{p}}_{\text{q}})\;\\ \text{=N }({\text{f}}_{0}{\text{p}}_{0}+\Sigma {\text{f}}_{\text{i}}{\text{R}}_{\text{i}}{\text{p}}_{0}+{\text{f}}_{\text{q}}{\text{R}}_{\text{q}}{\text{p}}_{0})\;\\ {\text{=N p}}_{0}({\text{f}}_{0}+\Sigma {\text{f}}_{\text{i}}{\text{R}}_{\text{i}}+{\text{f}}_{\text{q}}{\text{R}}_{\text{q}}) \end{array}$$

$$\begin{array}{l} {\text{PAF}}_{\text{i}}=\frac{\omega_{\text{i}}}{\text{M}}=\frac{{\text{N f}}_{\text{i}}({\text{R}}_{\text{i}}-1){\text{p}}_{0}}{{\text{Np}}_{0}({\text{f}}_{0}+\sum {\text{f}}_{\text{i}}\;{\text{R}}_{\text{i}}+{\text{f}}_{\text{q}}\;\text{​}\text{​}{\text{R}}_{\text{q}})}\\ =\frac{{\text{f}}_{\text{i}}({\text{R}}_{\text{i}}-1)}{{\text{f}}_{0}+\sum {\text{f}}_{\text{i}}\;{\text{R}}_{\text{i}}+{\text{f}}_{\text{q}}\;{\text{R}}_{\text{q}}} \end{array}$$

Using Allison's notations, the above equation can also be expressed as

$${\text{PAF}}_{\text{i}}=\frac{\text{P}({\text{O}}_{\text{i}})[{\text{RR}}_{\text{i}}-1]}{\text{P}(\text{R})+\sum \text{P}({\text{O}}_{\text{i}}){\text{RR}}_{\text{i}}+\text{P}(\text{Q}){\text{RR}}_{\text{q}}}$$

It then follows that

$$\begin{array}{l} \omega_{\text{i}}=\text{M}\;\cdot \;{\text{PAF}}_{\text{i}}\\ =\frac{\text{MP}({\text{O}}_{\text{i}})[{\text{RR}}_{\text{i}}-1]}{\text{P}(\text{R})+\sum \text{P}({\text{O}}_{\text{i}}){\text{RR}}_{\text{i}}+\text{P}(\text{Q}){\text{RR}}_{\text{q}}} \end{array}$$

Equations 11 and 12 are new equations we created for exposure category-specific PAF and attributable number, respectively. Because the Mokdad procedure uses the PAF approach, the neutral category Q reappears in equations 11 and 12, because Q is part of the total population. P(Q) is easy to obtain from health surveys. Equation 12 is expected to yield identical results as those of equation 10 (Allison's), because it is derived from equation 10. Equation 12 is simpler to use as it needs only total deaths (M), fractions of exposure in each category ((P(O_i_), P(R), P(Q)) which are readily available from health surveys and the relative risks (RR_i_, RR_q_). Additionally, equation 10 is difficult to use because it also requires total population (N), probability of death in each of the exposure categories (P(D|O_i_)), and probability of death in the reference group (P(D|R)). The probabilities of death are difficult to obtain.

### 4. Difference in the estimated number of attributable deaths under the hazard ratio and the relative risk models

Relative risk (RR) is an estimate of hazard ratio (HR). HR is the ratio of hazard rates (instantaneous incidence rates) in the exposed to the nonexposed at a point in time [4]. RR is the ratio of average risks of disease or death in the exposed to the nonexposed over a period of time.

$$\text{RR}=\frac{1-{\text{e}}^{-\text{h}\lambda }}{1-{\text{e}}^{-\lambda }}$$

where h is hazard ratio (HR) and λ is hazard rate in nonexposed.

In theory, as pointed out by Allison et al, RR estimates "without adjustment for time can bias results (though the bias may be small)" [1]. The question is, in practice, does it matter whether RR or HR is used? Table 5 is a theoretical comparison of HR and RR using equation 13, based on hazard rates of 0.01 and 0.10, and HR of 1, 3, 5, 7. When hazard rate is low (e.g., 0.10 or below), HR and RR are close to each other. The lower the hazard rate (e.g., 0.01), the closer together the HR and RR. From the real data for Alameda County Health Study provided by Allison et al, when HR was 1.39, the RR was 1.38766116; when HR was 0.98, the RR was 0.98008466 (Table 5). There is no practical difference in the real setting in the estimated number of attributable deaths under the HR and RR models.

**Table 5:** Comparison of hazard ratio (HR) and relative risk (RR) using the notations of Allison et al [1].

Hazard rate in nonexposed λ	Hazard ratio HR = h	Probability of death in nonexposed **P(D|R) = 1 - e**^**-λ**^	Probability of death in exposed **P(D|O) = 1 -e**^**-hλ**^	Relative risk $RR=\frac{1-{\text{e}}^{-h\lambda }}{1-{\text{e}}^{-\lambda }}$
Theoretical comparison				
				
0.01	1	0.00995	0.00995	1.00
	3		0.02955	2.97
	5		0.04877	4.90
	7		0.06761	6.79
				
0.10	1	0.09516	0.09516	1.00
	3		0.25918	2.72
	5		0.39346	4.13
	7		0.50341	5.29

Real example from table 3 of Allison et al [1]				
				
0.008651	1.39	0.00861368777	0.0119528799	1.38766116
				
0.008651	0.98	0.00861368777	0.0084421433	0.98008466

### 5. Options for numerically solving an equation for the hazard of death in the nonexposed (λ)

There are commercial packages available for solving an equation for an unknown quantity; packages such as MATHEMATICA and MAPLE [5]. However, for these packages there is a steep learning curve for beginners, and packages can be quite expensive [6]. We looked into two simpler non-commercial options which one can easily program at no cost.

The first option is Newton's method (Additional file 1, Appendix S2). Applying Newton's method to the Alameda County Health Study data, provided in Allison et al's table three, gave an estimated λ of 0.008651. The second option is Taylor series (Additional file 1, Appendix S3). Applying Taylor series to the same data gave the same estimated λ of 0.008651. The two options gave virtually the same answers, with an error margin of less than 0.000001.

### 6. Comparison of the Allison procedure and modified Mokdad procedure with real datasets

We used the real dataset from the Alameda County Health Study provided by Allison et al [1] to compare the results using the Allison procedure and the modified Mokdad procedure, under both the hazard ratio (HR) and the relative risk (RR) models (Additional file 1, Appendix S4).

From our Additional file 1, Appendix S4, it can be seen that the results using the Allison procedure and the modified Mokdad procedure, under the hazard ratio (HR) and the relative risk (RR) models, are very similar to each other. The Allison procedure is a HR approach and the Mokdad procedure is a RR approach. Therefore the results of the Mokdad procedure using RR are closer to the Allison procedure than the Mokdad procedure using HR. However, the Mokdad procedure using HR to approximate RR provides good enough estimates of attributable number of deaths, and it avoids the use of equation 13 which involves estimation of RR that involves complex estimation of λ.

## Discussion

The procedures recommended by Allison et al [1] and Mokdad et al [2] can both be applied to estimate the number, as well as fraction, of a single outcome (such as death) attributable to a risk factor (such as increased body mass index, BMI) that is polytomous (e.g., overweight, obese, and even stratified by BMI unit). Although not specifically mentioned in the two original articles [1,2], both procedures can be applied to one or more risk factor combinations (such as BMI and smoking) as long as the risk factor combinations are expressed in independent (i.e., nonoverlapping) exposure categories. Furthermore, both procedures are under the assumptions of no confounding and no effect modification by the risk factors of interest and other covariates (such as age or sex).

The Allison procedure can be applied to the situation when there is a nonexposed category, one or more exposure categories, and one or more neutral (neither nonexposed nor exposed) categories. Allowance of a neutral exposure category is a benefit of the Allison procedure from a causal inference perspective, because in reality the population cannot always be dichotomized into nonexposed and exposed. The Mokdad procedure cannot allow for a neutral category. This paper proposes a modified Mokdad procedure that can achieve the same results as the Allison procedure, but through a simpler way.

The Allison procedure involves twelve steps, while the Mokdad procedure involves only six steps (Additional file 1, Appendix S1). The reason why the Allison procedure involves more steps is because it attempts to directly estimate the attributable number of deaths (equation A1), and this necessitates the estimation of the probabilities of death in the nonexposed, various exposure and the neutral categories. This in turn necessitates the calculation of the hazard rate in the nonexposed, λ, which requires substantial mathematical skills. The Mokdad procedure, on the other hand, first calculates the population attributable fraction (equation A3), and then obtain the attributable number of deaths by multiplying the PAF with the total number of deaths (equation A2). Our paper (equations 1-4) shows that in the derivation of the equation for PAF in the Mokdad procedure, the probabilities of death cancel out each other in the numerator and denominator, leaving only fractions of exposure and relative risks as necessary input parameters for the estimation of PAF. This greatly simplifies the calculation process in the Mokdad procedure.

The Mokdad procedure, however, breaks down if a neutral category (such as underweight) that is neither nonexposed (such as normal weight) nor exposed (such as overweight and obese) exists. Also, while the Mokdad procedure can calculate the overall number of deaths attributable to a risk factor with multiple exposure categories, it does not calculate the number attributable to each individual exposure category. The Allison procedure, on the other hand, can estimate the individual exposure category attributable numbers (although the exact equation was not given in Allison et al [1].)

By combining the innovation of the Allison procedure [1] (i.e., allowing for a neutral category which is neither nonexposed nor exposed) and the simplicity of the Mokdad procedure [2] (i.e., calculating attributable numbers indirectly through population attributable fractions), this paper proposes a new procedure (modified Mokdad) to calculate population attributable fractions and numbers (Table 6). This paper also "recovers" the missing equation in Allison et al that calculates the individual exposure category attributable numbers (equation 10), and develops a similar equation using the Mokdad et al approach (equation 12). Furthermore, this paper extends the concept and equation of population attributable fraction, originally proposed by Levin [3], from a two-category risk factor (nonexposed, exposed) (equation 1) to a three-category risk factor (nonexposed, exposed, and neutral) (equation 8).

**Table 6:** Eight steps in our proposed new procedure (modified Mokdad) to calculate number of deaths attributable to a risk factor with multiple exposure categories, allowing for one or more neutral categories.

Let ω be the number of deaths attributable to a risk factor (e.g. overweight and obese); and ω_i _be the number of deaths attributable to a specific exposure category i (e.g. overweight) of the risk factor.
$$\omega =\Sigma \omega_{\text{i}}$$
$$\omega_{\text{i}}=\text{M}\cdot {\text{PAF}}_{\text{i}}$$
Using the notations in Table 2, ${\text{PAF}}_{\text{i}}=\frac{{\text{f}}_{\text{i}}({\text{R}}_{\text{i}}-1)}{{\text{f}}_{0}+\sum {\text{f}}_{\text{i}}{\text{R}}_{\text{i}}+{\text{f}}_{\text{q}}{\text{R}}_{\text{q}}}$
Using the notations of Allison et al, equation 11 can be expressed as ${\text{PAF}}_{\text{i}}=\frac{\text{P}({\text{O}}_{\text{i}})[{\text{RR}}_{\text{i}}-1]}{\text{P}(\text{R})+\sum \text{P}({\text{O}}_{\text{i}}){\text{RR}}_{\text{i}}+\text{P}(\text{Q}){\text{RR}}_{\text{q}}}$
Using the notations of Mokdad et al, and adding P_q _(fraction of population underweight) and RR_q _(relative risk for underweight), equation 11 becomes ${\text{PAF}}_{\text{i}}=\frac{{\text{P}}_{\text{i}}[{\text{RR}}_{\text{i}}-1]}{{\text{P}}_{0}+\sum {\text{P}}_{\text{i}}{\text{RR}}_{\text{i}}+{\text{P}}_{\text{q}}{\text{RR}}_{\text{q}}}$

Eight steps to estimate ω:

Step 1: Obtain M, total no. of deaths in the population during 1 y (from death records).

Step 2: Obtain f_0 _[or P(R) or P_0_], percentage of individuals in the population nonexposed (e.g. normal weight; never smokers) (from health surveys).

Step 3: Obtain f_i _[or P(O_i_) or P_i_], percentage in separate exposure categories of the risk factor (e.g. overweight or obese; current occasional smokers or current daily smokers) (from health surveys).

Step 4: Obtain f_q _[or P(Q) or P_q_], percentage in a neutral category (e.g. underweight; former smokers) (from health surveys).

Step 5: Obtain R_i _[or RR_i_], relative risk of death for each separate exposure category (e.g. former smokers, current smokers) relative to none (e.g. never smokers) (from prospective cohort studies). When R_i _is not available and when the event (e.g. death) is rare, relative risk can be approximated by HR (ie, h and q).

Step 6: Calculate PAF_i_, population attributable fraction, using equation 11.

Step 7: Calculate ω_i _using equation T5.

Step 8: Calculate ω using equation T4.

Both our proposed procedure (Table 6) and the Allison procedure allow for one or more neutral categories (such as underweight). The numbers of death associated with the neutral categories are excluded from the calculation of the number of death attributable to the risk factor under study. The Allison procedure involves twelve steps while the proposed procedure involves only eight steps. The proposed procedure, using the logic of the Mokdad procedure, does not require calculation of the probabilities of death in the various categories. Therefore no solving for the hazard λ is required. The proposed procedure is expected to produce similar results as the more complex Allison procedure. Slight discrepancies in the results, as shown in the real examples provided in this paper, are due to rounding errors in the additional steps in the Allison procedure to estimate probabilities of death in various nonexposed, exposure and neutral categories, and solving for λ, the hazard in the nonexposed, all of which are not required in the proposed procedure. Discrepancies will also occur depending on whether relative risks or hazard ratios are used, but this is expected to be small when the event (e.g., death) is rare (section 4). If one insists to use the Allison procedure instead of the proposed procedure, this paper discusses a number of options for solving for λ which could be helpful (section 5).

## Competing interests

The authors declare that they have no competing interests.

## Acknowledgements

The author would like to thank Sunita Narang, Justin Francis and Rita Zhang for statistical and data support for this paper. The author declares there is no conflict of interest. No funding or support was received for this study. The author has full access to all of the data in the study and takes responsibility for the integrity of the data and the accuracy of the data analysis.

## Supplementary Material

Additional file 1 **file containing Appendices S1-S4 **[7-10]. Click here for file
