# Supplementary material for: Population attributable fraction: comparison of two mathematical procedures to estimate the annual attributable number of deaths
Source: Epidemiol Perspect Innov. 2010 Aug 31;7:8. doi: 10.1186/1742-5573-7-8 (PMC2944136; doi:10.1186/1742-5573-7-8)
Supplement: Additional file 1 — file containing Appendices S1-S4 [7-10]. [file 1742-5573-7-8-S1.DOC]

**Additional file 1**

**Appendix S1. A comparison of the Allison et al procedure and Mokdad et al procedure**

The Allison procedure requires 12 steps (Table A1) and Mokdad procedure only 6 steps (Table A2). The reason is that there is a basic difference in the approach and logic of the two procedures. The Allison procedure directly estimates ω, the number of deaths attributable to an exposure (equation A1). This requires estimation of the probabilities of death in the nonexposed (p_0_ or P(D|R)) and underweight (p_q_ or P(D|Q)) categories. Because P(D|R) = 1 – e^-λ^ and P(D|Q) = 1 – e^-qλ^ this in turn leads to a requirement to estimate hazard of death in the nonexposed, λ, and λ can only be obtained by numerically solving a complex equation (Step 9 in Table A1).

On the other hand, the Mokdad procedure starts with estimating PAF, and then estimates ω indirectly by multiplying PAF with the total number of deaths, M (equation A2). By using PAF, the probability of death in nonexposed (p_0_) cancels each other out in the numerator and denominator (compare equations 3 and 4 in the text), and therefore PAF can be estimated simply from fractions of exposure (P_0_, P_i_) and relative risks (RR_i_) (see equation A3).

Table A1. Twelve steps in the Allison Procedure to calculate number of deaths attributable to a risk factor such as obesity [1].

|  |
| --- |
| Let ω be the number of deaths attributable to obesity or overweight, ie, the number of individuals in a given year who are expected to die who would not be expected to die if all obese or overweight persons had the same hazard as the average person in the reference category.  ω = M – N[P(R)P(D|R) + P(O)P(D|R) + P(Q)P(D|Q)] … (A1) |
|  |
| Twelve steps to estimate ω:  Step 1: Obtain M, total no. of deaths among individuals 18 y or older expected to occur in the population during 1 y (2 110 687 in US in 1991 from death records).  Step 2: Obtain N, total no. of people 18 y or older in the population (185 105 441 in US in 1991 from census).  Step 3: Obtain P(R), probability of membership in reference (ie, nonoverweight and nonunderweight) category (from health surveys).  Step 4: Obtain P(O), probability of obesity or overweight (from health surveys).  Step 5: Obtain P(Q), probability of nonobesity and nonmembership in reference category (from health surveys).  Step 6: Obtain h, the hazard ratio (HR) for an obese or overweight person compared with a person in the reference category (from prospective cohort studies).  Step 7: Obtain q, the HR for a nonobese person who is also not in the reference category compared with someone in the reference category (from prospective cohort studies).  Step 8: Calculate P(D), probability of a member of the population dying in 1 y, where P(D) = M/N.  Step 9: Calculate λ, the hazard for a person in the reference category. Can be obtained by numerically solving the following equation for λ:   1 − P(D) = P(O)e^-hλ^ + P(R)e^-λ^ + (1-P(O)-P(R))e^-qλ^  Step 10: Calculate P(D|R), probability of a member of the population dying in 1 y given that he/she is in the reference category, where P(D|R) = 1 – e^-λ^  Step 11: Calculate P(D|Q), probability of a member of the population dying in 1 y given that he/she is not in either the obese or overweight category or the reference category, where P(D|Q) = 1 – e^-qλ^  Step 12: Calculate ω using equation A1. |
|  |

Table A2. Six steps in the Mokdad Procedure to calculate number of deaths attributable to a risk factor such as smoking [2].

|  |
| --- |
| Let ω be the number of deaths attributable to a risk factor. |
|  |
| ω = M  PAF … (A2) |
|  |
| PAF = {[P_0_ +  P_i_(RR_i_)] -1} / [P_0_ +  P_i_(RR_i_)] … (A3) |
|  |
| Six steps to estimate ω:  Step 1: Obtain M, total no. of deaths among individuals 18 y or older expected to occur in the population during 1 y (from death records).  Step 2: Obtain P_0_, percentage of individuals in the population not engaging in the risk behaviour (e.g. never smokers) (from health surveys). [P_0_ is the same as P(R).]  Step 3: Obtain Pi, percentage engaging in separate categories of the risk behaviour (e.g. former smokers, current smokers) (from health surveys). [P_i_ is similar to P(O) and P(Q).]  Step 4: Obtain RR_i_, relative risk of death for each separate category (e.g. former smokers, current smokers) relative to none (e.g. never smokers) (from prospective cohort studies). [RR_i_ is numerically similar to HR (ie, h and q) when the event is rare.]  Step 5: Calculate PAF, population attributable fraction, using equation A3.  Step 6: Calculate ω using equation A2. |
|  |

**Appendix S2. Newton’s method for numerically solving for the hazard of death in the nonexposed (λ)**

Newton’s method, also called Newton-Raphson iteration, is a root-finding algorithm [7,8]. It finds successively through iterations better approximations to the roots of a real-valued function. To use this method, one must first consider a nonlinear equation, defined as f(x) = 0. To find its roots (i.e. values of x where the equation is satisfied), one starts with an initial (blind) guess of the root (call it x_0_), then the function is approximated by its [tangent line](http://en.wikipedia.org/wiki/Tangent_line). One then computes the x-intercept of this tangent line, which is a better approximation to the function's root than the initial guess, and the method is [iterated](http://en.wikipedia.org/wiki/Iterative_method).

Newton’s method cannot be used in all cases but, fortunately, the particular equation put forward by Allison et al (A4) is relatively easy to solve for λ using Newton’s Method. The derivatives of the equation to be solved are all defined, continuous and easy to compute. From Allison et al [1],

1 − P(D) = P(O)e^-hλ^ + P(R)e^-λ^ + (1-P(O)-P(R))e^-qλ^

= P(O)e^-hλ^ + P(R)e^-λ^ + P(Q)e^-qλ^ … (A4)

f(λ) = P(O)e^-hλ^ + P(R)e^-λ^ + P(Q)e^-qλ^ + P(D) − 1 = 0

f’(λ) = - hP(O)e^-hλ^ − P(R)e^-λ^ − q P(Q)e^-qλ^

Let the initial guess be λ_0_. Solve for f(λ_0_) and f’(λ_0_) where f’ is the first derivative of f(λ) computed for λ = λ_0_.  Then, according to Newton’s method, a better guess for λ, is λ_1_ = λ_0_ + δ_0_ where δ_0_ = - f(λ_0_)/f’(λ_0_). Iterate (i.e. repeat the above process) with each new guess for λ (i.e. λ_0_, λ_1_, etc) until the correction δ_λ_ is sufficiently small.

$$\lambda_{\text{n}+1}\text{ }=\lambda_{n}+\delta_{n}=\lambda_{n}-\frac{\text{f(}\lambda_{\text{n}})}{f'(\lambda_{n})}$$

We used an Excel spreadsheet to carry out Newton’s method to solve for λ. In the following spreadsheet (Table A3), the parameters are obtained from Allison et al [1], table 3 for Alameda County Health Study, but with one important typographical error corrected – the correct value for P(Q) is 0.290 not 0.291. Use of the incorrect value has led to very different numbers of attributable deaths from those reported in Allison et al. P(D), probability of death in a year, is M/N = 2 110 687 / 185 105 441 = 0.011403. λ_0 is our initial guess of the root. The Excel equations that we used are:

λ: E4 = E3+H3

f(λ): F3 = P_O1*EXP(-h_1*E3)+P_O2*EXP(-h_2*E3)+P_O3*EXP(-h_3*E3)+P_O4*EXP(-h_4*E3)+P_O5*EXP(-h_5*E3)+P_O6*EXP(-h_6*E3)+P_O7*EXP(-h_7*E3)+P_R*EXP(-E3)+P_Q*EXP(-q*E3)+P_D-1

f'(λ): G3 = -h_1*P_O1*EXP(-h_1*E3) - h_2*P_O2*EXP(-h_2*E3) - h_3*P_O3*EXP(-h_3*E3) - h_4*P_O4*EXP(-h_4*E3) - h_5*P_O5*EXP(-h_5*E3) - h_6*P_O6*EXP(-h_6*E3) - h_7*P_O7*EXP(-h_7*E3) - P_R*EXP(-E3) - q*P_Q*EXP(-q*E3)

δ: H3 = -F3/G3

The solution for λ for Alameda County Health Study is 0.008651. Newton's method can often converge remarkably quickly, especially if the iteration begins "sufficiently near" the desired root. Just how near "sufficiently near" needs to be, and just how quickly "remarkably quickly" can be, depends on the problem. In our example given here, when the initial guess is 2, it takes 22 iterations, but when the initial guess is 1, it takes only 7 iterations, both leading to exactly the same answer (0.008651).

Table A3. Excel spreadsheet to carry out Newton’s method to solve for λ (x-value).

|  | A | B | C | D | E | F | G | H |
| --- | --- | --- | --- | --- | --- | --- | --- | --- |
| 1 |  |  |  |  |  |  |  |  |
|  |  |  |  |  |  |  |  |  |
| 2 | Parameters | |  | index | λ | f(λ) | f'(λ) | δ |
|  |  |  |  |  |  |  |  |  |
| 3 | H1= | 0.98 |  | 0 | 2 | -0.89873 | -0.10178 | -8.82971 |
|  |  |  |  |  |  |  |  |  |
| 4 | H2= | 0.86 |  | 1 | -6.82971 | 14525053 | -4.1E+07 | 0.358504 |
|  |  |  |  |  |  |  |  |  |
| 5 | H3= | 1.20 |  | 2 | -6.47121 | 5343843 | -1.5E+07 | 0.358559 |
|  |  |  |  |  |  |  |  |  |
| 6 | H4= | 1.26 |  | 3 | -6.11265 | 1966123 | -5481979 | 0.358652 |
|  |  |  |  |  |  |  |  |  |
| 7 | H5= | 1.23 |  | 4 | -5.754 | 723440.5 | -2016228 | 0.358809 |
|  |  |  |  |  |  |  |  |  |
| 8 | H6= | 1.36 |  | 5 | -5.39519 | 266228.6 | -741431 | 0.359074 |
|  |  |  |  |  |  |  |  |  |
| 9 | H7= | 2.79 |  | 6 | -5.03611 | 97995.85 | -272572 | 0.359523 |
|  |  |  |  |  |  |  |  |  |
| 10 | Q= | 1.39 |  | 7 | -4.67659 | 36085.41 | -100159 | 0.360282 |
|  |  |  |  |  |  |  |  |  |
| 11 | P(D)= | 0.011403 |  | 8 | -4.31631 | 13296.71 | -36774.9 | 0.36157 |
|  |  |  |  |  |  |  |  |  |
| 12 | P(O1)= | 0.083 |  | 9 | -3.95474 | 4905.023 | -13484.6 | 0.36375 |
|  |  |  |  |  |  |  |  |  |
| 13 | P(O2)= | 0.076 |  | 10 | -3.59099 | 1812.773 | -4933.65 | 0.36743 |
|  |  |  |  |  |  |  |  |  |
| 14 | P(O3)= | 0.065 |  | 11 | -3.22356 | 671.9825 | -1798.67 | 0.3736 |
|  |  |  |  |  |  |  |  |  |
| 15 | P(O4)= | 0.052 |  | 12 | -2.84996 | 250.2834 | -652.13 | 0.383794 |
|  |  |  |  |  |  |  |  |  |
| 16 | P(O5)= | 0.045 |  | 13 | -2.46617 | 93.8656 | -234.601 | 0.400108 |
|  |  |  |  |  |  |  |  |  |
| 17 | P(O6)= | 0.142 |  | 14 | -2.06606 | 35.50389 | -83.6621 | 0.424372 |
|  |  |  |  |  |  |  |  |  |
| 18 | P(O7)= | 0.077 |  | 15 | -1.64168 | 13.5082 | -29.752 | 0.454026 |
|  |  |  |  |  |  |  |  |  |
| 19 | P(R)= | 0.170 |  | 16 | -1.18766 | 5.083824 | -10.8311 | 0.469374 |
|  |  |  |  |  |  |  |  |  |
| 20 | P(Q)= | 0.290 |  | 17 | -0.71828 | 1.795601 | -4.31561 | 0.416071 |
|  |  |  |  |  |  |  |  |  |
| 21 | λ_0= | 2 |  | 18 | -0.30221 | 0.520843 | -2.11642 | 0.246096 |
|  |  |  |  |  |  |  |  |  |
| 22 |  |  |  | 19 | -0.05612 | 0.089059 | -1.44288 | 0.061723 |
|  |  |  |  |  |  |  |  |  |
| 23 |  |  |  | 20 | 0.005605 | 0.003998 | -1.31562 | 0.003039 |
|  |  |  |  |  |  |  |  |  |
| 24 |  |  |  | 21 | 0.008644 | 9E-06 | -1.3097 | 6.87E-06 |
|  |  |  |  |  |  |  |  |  |
| 25 |  |  |  | 22 | 0.008651 | 4.59E-11 | -1.30968 | 3.51E-11 |
|  |  |  |  |  |  |  |  |  |
| 26 |  |  |  | 23 | 0.008651 | 0 | -1.30968 | 0 |
|  |  |  |  |  |  |  |  |  |
| 27 |  |  |  | 24 | 0.008651 | 0 | -1.30968 | 0 |
|  |  |  |  |  |  |  |  |  |
| 28 |  |  |  | 25 | 0.008651 | 0 | -1.30968 | 0 |
|  |  |  |  |  |  |  |  |  |

**Appendix S3. Taylor series for numerically solving for the hazard of death in the nonexposed (λ)**

The Taylor series is a representation of a [function](http://en.wikipedia.org/wiki/Function_(mathematics)) as an [infinite sum](http://en.wikipedia.org/wiki/Series_(mathematics)) of terms calculated from the values of its [derivatives](http://en.wikipedia.org/wiki/Derivative) at a single point, a [9,10].

$$\text{f(x)}=\text{f(a)}+\text{f'(a)(x-a)}+\frac{\text{f"(a)}}{\text{2!}}\text{(x-a)}^{2}+\frac{\text{f}^{\text{(3)}}(a)}{3!}(x-a)^{3}+...+\frac{f^{(n)}(a)}{n!}(x-a)^{n}+....$$

If the series uses the derivatives at zero (a=0), the series is also called a Maclaurin series. The Maclaurin series is computationally much simpler to use. It is valid here because we are working under the rare “event” assumption and the hazard λ will always be small (close to zero).

From Allison et al [1],

1 − P(D) = P(O)e^-hλ^ + P(R)e^-λ^ + P(Q)e^-qλ^ … (A4)

Let E_i_ = O_i_ for i = 1, …, I-2; E_I-1_ = R; E_I_ = Q; a_i_ = h_i_ for i = 1, …, I-2; a_I-1_ = 1; a_I_ = q

Then we can rewrite equation A4 as

$$\text{1 - P(D) }=\sum_{i=1}^{I} P(E_{i})e^{-a_{i}\lambda}$$

Using Maclaurin series expansion,

$$\text{1 - P(D) }=\sum_{i=1}^{I} P(E_{i})\left[\sum_{n=0}^{\infty} \frac{(-a_{i})^{n}\lambda^{n}}{n!}\right]=\sum_{n=0}^{\infty} \left[\sum_{i=1}^{I} P(E_{i})(-a_{i})^{n}\right]\frac{\lambda^{n}}{n!}$$

Then, using a second degree Maclaurin polynomial (which are partial sums of the Maclaurin series to the second degree) instead of an infinite series, we get

$$\text{1 - P(D) }\approx\sum_{i=1}^{I} P(E_{i})+\sum_{i=1}^{I} P(E_{i})(-a_{i})\lambda+\sum_{i=1}^{I} \frac{P(E_{i})(-a_{i})^{2}\lambda^{2}}{2}$$

Since $\sum_{i=1}^{I} P(E_{i})=1$, this is equivalent to

$$\text{ }\sum_{i=1}^{I} \frac{P(E_{i})(a_{i})^{2}\lambda^{2}}{2}-\sum_{i=1}^{I} P(E_{i})(a_{i})\lambda+P(D)\approx0$$

So we can approximate λ by

$\lambda^{\wedge} \text{ }=\frac{\sum_{i=1}^{I} P(E_{i})(a_{i})-\sqrt{\left(\sum_{i=1}^{I} P(E_{i})(a_{i})\right)^{2}-2P(D)\left(\sum_{i=1}^{I} P(E_{i})(a_{i})^{2}\right)}}{\sum_{i=1}^{I} P(E_{i})(a_{i})^{2}}$ … (A5)

In theory there are two quadratic roots for equation A5, corresponding to plus or minus the square root term. But in reality, $\lambda^{\wedge}$ is always the quadratic root as given in A5; the other quadratic root is not used. The mathematical proof is available from the author on request.

Again using the data for Alameda County Health Study from Allison et al [1] (see Appendix 2) with the correct value for P(Q) of 0.290, λ is estimated to be 0.008651 using the method of Taylor series (equation A5).

We used an Excel spreadsheet to carry out Taylor series to solve for λ. In the following spreadsheet (Table A4) there are very few steps involved. The column P(Ei)*ai is a weighted sum of the hazards, and the column P(Ei)*ai^2 is a weighted sum of squares of the hazards. Lambda is calculated using only these two sums and P(D). When the correct P(Q) of 0.290 is used, λ is 0.008651 as expected.

The Excel equations that we used are:

P(Ei)*ai: F3 = B3*D3

P(Ei)*ai^2: G3 = B3*F3

SUM P(Ei)*ai: F13 = SUM(F3:F11)

SUM P(Ei)*ai^2: G13 = SUM(G3:G11)

λ: F15 = (F13 - SQRT(F13^2 - 2*P_D*G13))/G13

Table A4. Excel spreadsheet to carry out Taylor series to solve for λ using a 2nd degree Maclaurin (Taylor) series.

|  | A | B | C | D | E | F | G |
| --- | --- | --- | --- | --- | --- | --- | --- |
| 1 |  |  |  |  |  |  |  |
|  |  | | | | | | |
| 2 | Parameters |  |  |  |  | P(Ei)*ai | P(Ei)*ai^2 |
|  |  |  |  |  |  |  |  |
| 3 | h1= | 0.98 | P(O1)= | 0.083 |  | 0.08134 | 0.079713 |
|  |  |  |  |  |  |  |  |
| 4 | h2= | 0.86 | P(O2)= | 0.076 |  | 0.06536 | 0.05621 |
|  |  |  |  |  |  |  |  |
| 5 | h3= | 1.20 | P(O3)= | 0.065 |  | 0.078 | 0.0936 |
|  |  |  |  |  |  |  |  |
| 6 | h4= | 1.26 | P(O4)= | 0.052 |  | 0.06552 | 0.082555 |
|  |  |  |  |  |  |  |  |
| 7 | h5= | 1.23 | P(O5)= | 0.045 |  | 0.05535 | 0.068081 |
|  |  |  |  |  |  |  |  |
| 8 | h6= | 1.36 | P(O6)= | 0.142 |  | 0.19312 | 0.262643 |
|  |  |  |  |  |  |  |  |
| 9 | h7= | 2.79 | P(O7)= | 0.077 |  | 0.21483 | 0.599376 |
|  |  |  |  |  |  |  |  |
| 10 | ref= | 1.00 | P(R)= | 0.170 |  | 0.17 | 0.17 |
|  |  |  |  |  |  |  |  |
| 11 | q= | 1.39 | P(Q)= | 0.290 |  | 0.4031 | 0.560309 |
|  |  |  |  |  |  |  |  |
| 12 |  |  |  |  |  |  |  |
|  |  |  |  |  |  |  |  |
| 13 | P(D)= | 0.011403 |  |  | SUM= | 1.32662 | 1.972486 |
|  |  |  |  |  |  |  |  |
| 14 |  |  |  |  | P(D)= | 0.011403 |  |
|  |  |  |  |  |  |  |  |
| 15 |  |  |  |  | λ= | 0.008651 |  |
|  |  |  |  |  |  |  |  |

**Appendix S4. Comparison of the Allison procedure and the modified Mokdad procedure using equations provided in this paper, under the hazard ratio (HR) and relative risk (RR) models, with Alameda County Health Study data provided by table 3 of Allison et al [1].**

| **BMI Category, kg/m^2^** | **P(CAT)*** | **Hazard ratios (HR)^#^** | **Relative risks (RR)**  **(Equation 13)^^** | **No. of deaths attributable to obesity** | | |
| --- | --- | --- | --- | --- | --- | --- |
|  |  |  |  | **Allison procedure**  **Using HR**  **(Equation 10)^^** | **Modified Mokdad**  **procedure**  **Using HR as RR**  **(Equation 12)^^** | **Modified Mokdad**  **procedure**  **Using RR**  **(Equation 12)^^** |
|  |  |  |  |  |  |  |
| 25 to <26 | 0.083 | 0.98 | 0.98008466 | -2636 | -2641 | -2635 |
|  |  |  |  |  |  |  |
| 26 to <27 | 0.076 | 0.86 | 0.86052025 | -16902 | -16929 | -16901 |
|  |  |  |  |  |  |  |
| 27 to <28 | 0.065 | 1.20 | 1.19896397 | 20620 | 20683 | 20619 |
|  |  |  |  |  |  |  |
| 28 to <29 | 0.052 | 1.26 | 1.25858607 | 21440 | 21511 | 21439 |
|  |  |  |  |  |  |  |
| 29 to <30 | 0.045 | 1.23 | 1.22877889 | 16415 | 16467 | 16414 |
|  |  |  |  |  |  |  |
| 30-35 | 0.142 | 1.36 | 1.35788748 | 81030 | 81333 | 81026 |
|  |  |  |  |  |  |  |
| >35 | 0.077 | 2.79 | 2.76854000 | 217127 | 219291 | 217117 |
|  |  |  |  |  |  |  |
|  |  |  |  |  |  |  |
| Total |  |  |  | 337094 | 339716 | 337078 |

*Fraction of total population in each category. Obtained directly from Allison et al.

^#^ Obtained directly from Allison et al for Alameda County Health Study.

^^ Equations are listed below. For explanations see text.

ω_i_ = N P(O_i_) [P(D|O_i_) – P(D|R)] … (10)

ω_i_$\text{ }=\frac{\text{M P(O}_{\text{i}}\text{) [RR}_{\text{i}}-1]}{\text{P(R)}+\sum P(O_{i})RR_{i}+P(Q)RR_{q}}$ … (12)

RR = $\text{ }\frac{1-\text{e}^{\text{-h}\lambda}}{1-e^{\text{-}\lambda}}$ … (13)
